# Neuroendoscopic resection of pineal region tumors via the transfrontal-transventricular-transforaminal approach

**DOI:** 10.3389/fneur.2025.1710012

**Published:** 2025-11-19

**Authors:** Tengyun Guo, Kunlin Hou, Jinqian Dong, Wenzhen Yang, Zhijian Guo, Qiang Li

**Affiliations:** The Second Hospital of Lanzhou University, Lanzhou, Gansu Province, China

**Keywords:** foramen of Monro, neuroendoscopy, pineal region tumor, transventricular approach, tumor resection

## Abstract

**Purpose:**

To evaluate the clinical efficacy of neuroendoscopic resection of pineal region tumors via the transfrontal-transventricular-transforaminal approach.

**Methods:**

Clinical data of eight patients with pineal region tumors who underwent this surgical approach at our institution between January 2020 and July 2025 were retrospectively reviewed.

**Results:**

The cohort consisted of seven males and one female, aged 3–52 years. Gross total resection (GTR) was achieved in three patients, near-total resection (NTR) in two, and subtotal resection (STR) in three. Three patients underwent combined radiotherapy and chemotherapy. Histopathological diagnoses included three mixed germ cell tumor, two low-grade glioma, one high-grade glioma, and one mature teratoma. Two patients developed subjective memory decline. During a follow-up period of 3–29 months, seven patients resumed normal daily activities, except for one who discontinued treatment due to tumor recurrence.

**Conclusion:**

Neuroendoscopic resection of pineal region tumors via the transfrontal-transventricular-transforaminal approach is a safe and effective technique, particularly for lesions extending into the third ventricle.

## Introduction

Previous studies have shown that radical resection offers a better prognosis for nearly all patients with benign pineal region tumors and for most patients with malignant tumors (1). However, the pineal region is located deep along the midline of the brain, and the complexity of surrounding anatomical structures has long posed a significant challenge to surgical intervention in this area (2–4). In recent years, neuroendoscopic techniques have emerged as a promising approach for treating pineal region tumors, offering several advantages, including improved visualization, minimally invasive access, reduced blind spots, and high-resolution imaging (5, 6).

The neuroendoscopic transfrontal-transventricular-transforaminal approach follows a natural intracranial pathway, allowing entry into the third ventricle without disrupting adjacent neural or vascular structures. This approach is commonly used to manage lesions within and around the third ventricle (7–9). Anatomical studies have demonstrated that it provides excellent visualization of the pineal region while minimizing interference from the vein of Galen complex, highlighting its potential for effective tumor resection in this area (10–12).

In this study, we retrospectively analyzed the clinical data of eight patients who underwent neuroendoscopic resection of pineal region tumors using this transfrontal-transventricular-transforaminal approach to evaluate its therapeutic efficacy.

## Materials and methods

### Inclusion and exclusion criteria

Inclusion criteria: (1) Complete clinical data available; (2) Imaging demonstrating a space-occupying lesion in the pineal region combined with third ventriculomegaly; (3) Patients or their guardians fully understood the condition and consented to neuroendoscopic resection of the lesion via the transfrontal-transventricular-transforaminal approach.

Exclusion criteria: (1) Incomplete clinical data; (2) Prior relevant surgical treatment before admission (i.e., not initial surgery); (3) Unavailable postoperative prognosis data due to loss to follow-up or refusal of participation by patients or families.

### Clinical data

Eight patients who underwent neuroendoscopic resection of pineal region tumors via the transfrontal-transventricular-transforaminal approach at our institution from January 2020 to July 2025 were included. Informed consent was obtained from all patients and their families.

### Neuroimaging findings

All patients underwent preoperative plain and contrast-enhanced cranial MRI to localize the tumor within the pineal region and evaluate its relationship with adjacent structures, including the foramen of Monro, interthalamic adhesion, cerebral aqueduct, and deep venous system. All patients exhibited varying degrees of hydrocephalus before surgery.

### Surgical approach

**Figure 1:**
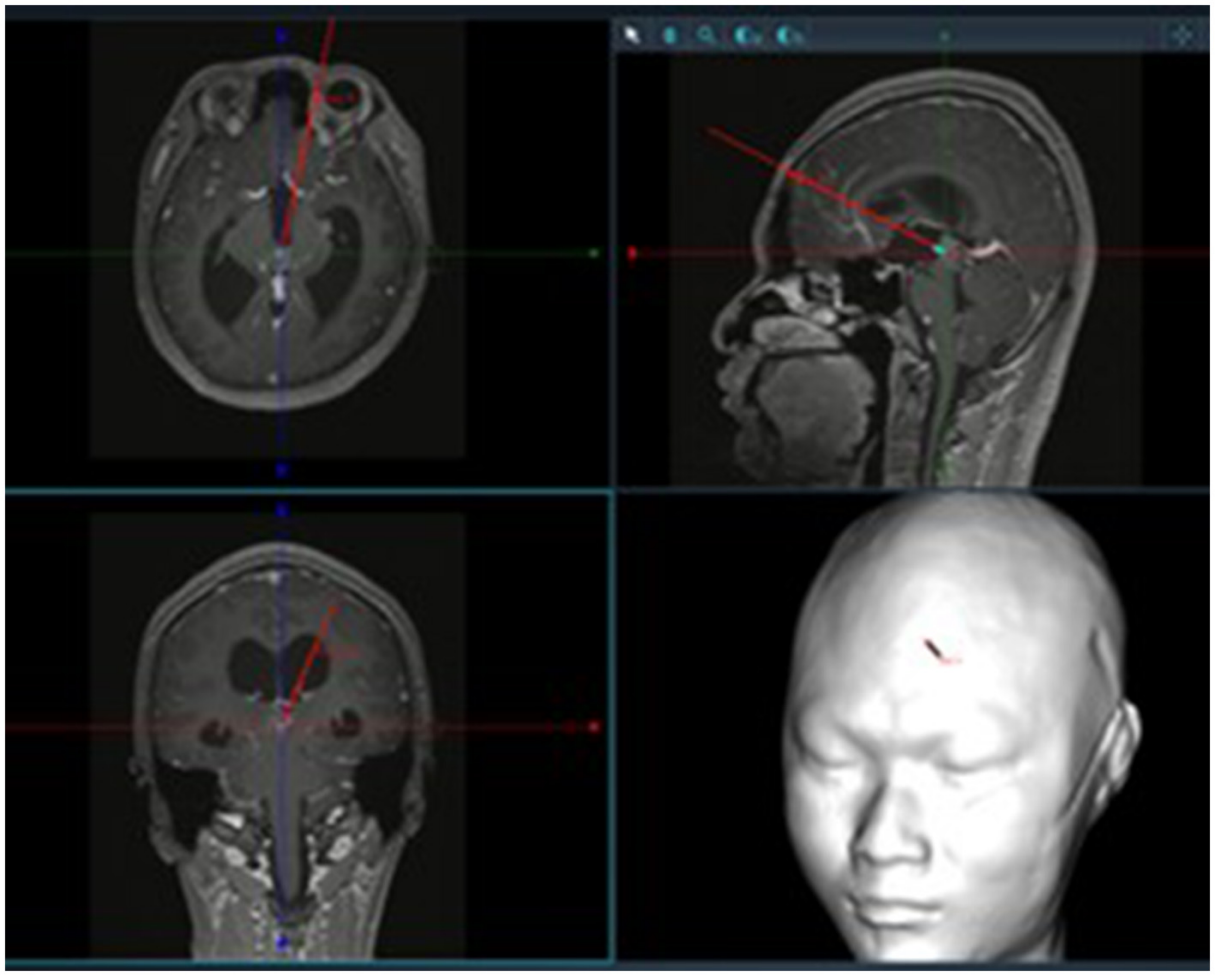
Surgical trajectory planning using neuronavigation.

**Figure 2:**
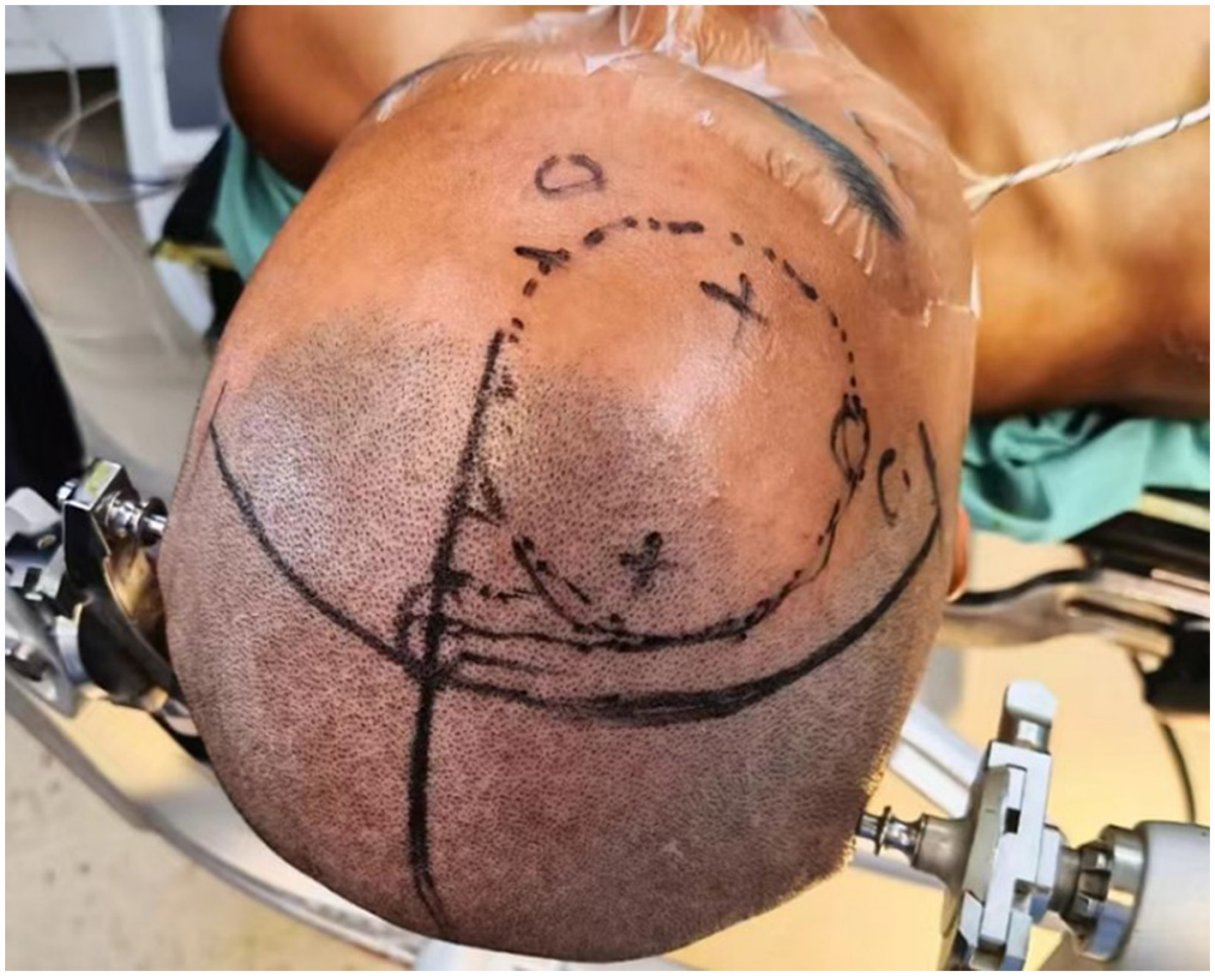
Localization of bone openings for tumor resection and third ventriculostomy (typically ~2.5 cm lateral to midline and ~5.2 cm above the eyebrow ridge for tumor resection; ~2.5 cm lateral and ~10.0 cm above the eyebrow ridge for ventriculostomy).

Patients were positioned supine with the head elevated 15°, fixed using a Mayfield head frame under general anesthesia. Burr hole locations were planned using the Huake Precision Neurosurgery Robot: (1) The trajectory for tumor resection was determined by projecting the line connecting the foramen of Monro and the anterior tumor margin to the frontal bone surface. (2) The burr hole for third ventricular floor fenestration was planned along the line from the foramen of Monro to the infundibular recess (Figures 1, 2). A right semi-coronal incision was made, and holes drilled under robotic navigation.

A pneumatic arm was used to stabilize the ventriculoscope (Karl Storz, Germany; 4 mm in diameter, 18 cm in length, 0° lens, with irrigation and working channels). Using an endoscopic access port, a puncture was made along the line connecting the external auditory canals to access the right lateral ventricle. The right foramen of Monro was identified at the intersection of the septal vein, thalamostriate vein, and choroid plexus.

The ventriculoscope was advanced through the foramen of Monro into the anterior third ventricle, exposing the anterior margin of the interthalamic adhesion, the choroid plexus extending from the lateral ventricle to the roof of the third ventricle, and the anterior commissure. The endoscope was further advanced posteriorly along the roof of the third ventricle, passing above the anterior commissure and the interthalamic adhesion, to expose the tumor, pineal recess, posterior commissure, and the opening of the cerebral aqueduct. A pistol-grip endoscopic scissor was introduced via the working channel. After confirming the absence of critical vessels, the tumor was resected piecemeal; tumor tissue was collected with forceps for intraoperative frozen pathology. Resection continued until complete tumor removal and patency of the aqueduct were confirmed. After meticulous hemostasis, the dura was sutured, the bone flap replaced, and the incision closed.

### Postoperative treatment

After obtaining the complete postoperative pathological report, multidisciplinary consultations involving radiation oncologists and medical oncologists were conducted. Adjuvant therapy (radiotherapy and/or chemotherapy) was formulated comprehensively based on the extent of tumor resection, pathological diagnosis, and the patient's general condition. During subsequent reexaminations and follow-up visits, the treatment regimens were dynamically adjusted according to the tumor's status (e.g., imaging evidence of tumor residue or recurrence) and changes in the patient's treatment tolerance.

### Follow-up

Follow-up combined clinical assessments and imaging. Functional status was evaluated clinically, and tumor recurrence was monitored by serial imaging. Telephone follow-ups were conducted at 1, 3, 6, and 12 months postoperatively, then every 6 months thereafter. Cranial imaging was recommended at 3, 6, and 12 months post-discharge, then annually.

### Statistical analysis

Descriptive statistics summarized baseline characteristics and clinical outcomes. Due to the small sample size, symptom improvement was assessed qualitatively, and proportions of symptom relief were calculated.

## Results

### Patient characteristics

A total of eight patients underwent pineal tumor resection using the transfrontal-transventricular-transforaminal approach. The cohort included seven males and one females, with a median age of 18.5 years (range, 3–52 years). Headache was the most common presenting symptom (6/8, 75%), followed by dizziness (4/8, 50%). One patient presented with gait instability, one patient with visual impairment, and one pediatric patient with vomiting, somnolence, and paroxysmal seizures.

Of the eight patients, two underwent preoperative external ventricular drainage due to acute intracranial hypertension. During surgery, three patients underwent endoscopic third ventriculostomy (ETV) (see Table 1).

**Table 1:** Basic clinical information, treatment details, and follow-up outcomes of patients included in the study.

**Sex/age**	**Preoperative symptoms**	**Tumor size (mm)**	**Preoperative hydrocephalus management**	**Extent of excision**	**Pathological diagnosis**	**Ostoperative treatment**	**Follow-up time (months)**	**Follow-up results**
M/15	H	36^*^15^*^25	NR	GTR	Mixed germ cell tumors	Pending	2	Recurrence
M/3	Vomiting, somnolence, episodic seizures	7^*^8^*^8	EVD	NTR	Mature teratoma	NR	29	Unremarkable
M/22	H/D	20^*^17^*^16	NR	GTR	Mixed germ cell tumors	NR	8	Unremarkable
M/52	H/D	28^*^18^*^24	NR	NTR	High-grade glioma	RT/ChT	6	memory decline
F/45	H/D, blurred vision	20^*^18^*^22	NR	STR	Low-grade glioma	NR	4	memory decline
M/51	H	13^*^20^*^15	NR	STR	Central neurocytoma	RT/ChT	5	Unremarkable
M/8	H	4^*^4^*^4	EVD	GTR	Mixed germ cell tumors	RT/ChT	5	Unremarkable
M/10	D, gait instability	16^*^16^*^16	NR	STR	Low-grade glioma	NR	3	Unremarkable

H, headaches; D, dizziness; NR, none required; EVD, external ventricular drainage; GTR, gross total resection; NTR, near-total resection; STR, subtotal resection; RT, radiotherapy; ChT, chemotherapy.

### Imaging characteristics, surgical findings, and pathology

Tumor diameter ranged from 4 to 36 mm, with a mean of 19.25 mm. Preoperative imaging confirmed third ventricle hydrocephalus in all patients. Postoperative MRI within 1 week demonstrated GTR in three patients, NTR in two, and STR in three. Postoperative imaging showed significant resolution of hydrocephalus in all patients, with no evidence of intracranial or surgical site hemorrhage. Histopathology revealed three mixed germ cell tumor, two low-grade glioma, one high-grade glioma, and one mature teratoma.

### Follow-up results

One patient developed episodic seizures 2 months postoperatively. Cranial CT on readmission revealed a new frontal lobe lesion; the family elected to discontinue treatment. Among the seven patients who completed follow-up, six achieved complete symptom relief (85.7%). Overall, symptom relief in the entire cohort was 75%. One patient who presented with preoperative dizziness, headache, and blurred vision experienced complete resolution of dizziness and headache, with partial improvement of visual blurring after surgery. Two patients reported postoperative memory decline compared with their preoperative status, but this did not affect their normal learning or daily activities. Due to follow-up limitations, memory impairment was not objectively assessed with standardized scales. All patients except the one who discontinued treatment due to tumor recurrence showed significant radiological improvement of hydrocephalus during the follow-up period. No venous sinus thrombosis or other long-term complications were observed in any patients. All seven patients maintained normal learning and daily functioning.

### Typical case

**Figure 3:**
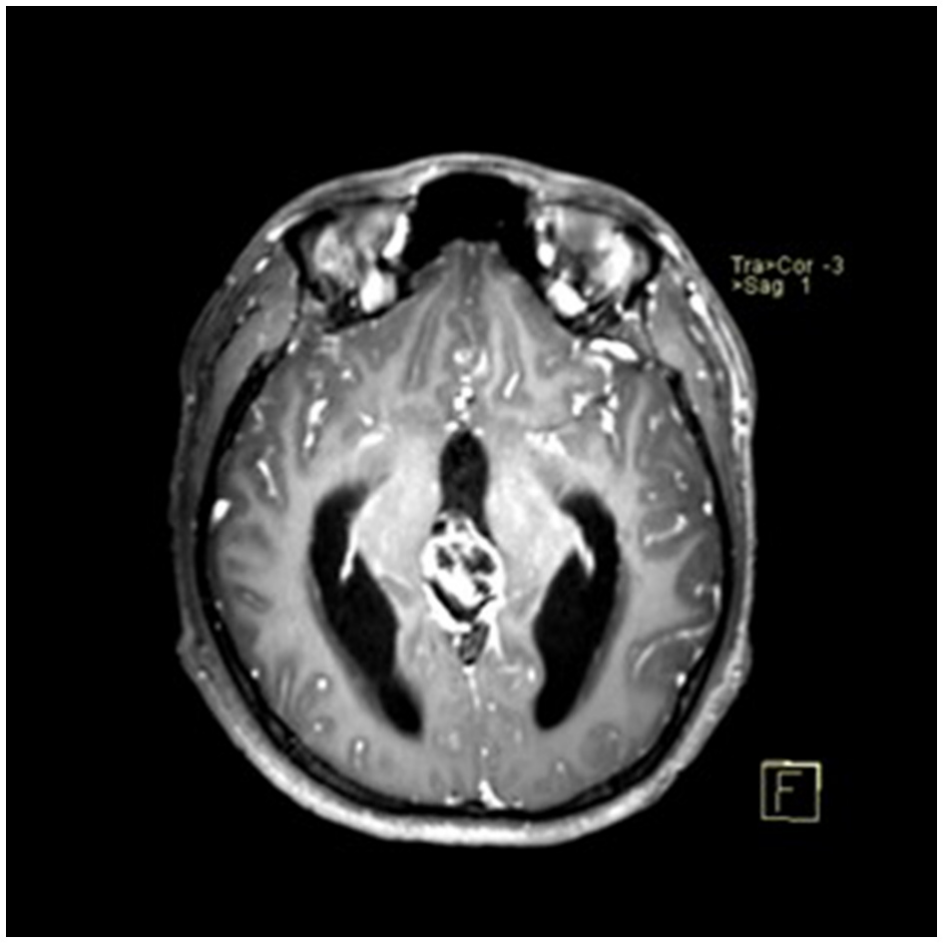
Preoperative contrast-enhanced MRI (axial view) showing a cystic-solid mass in the pineal region with supratentorial hydrocephalus.

**Figure 4:**
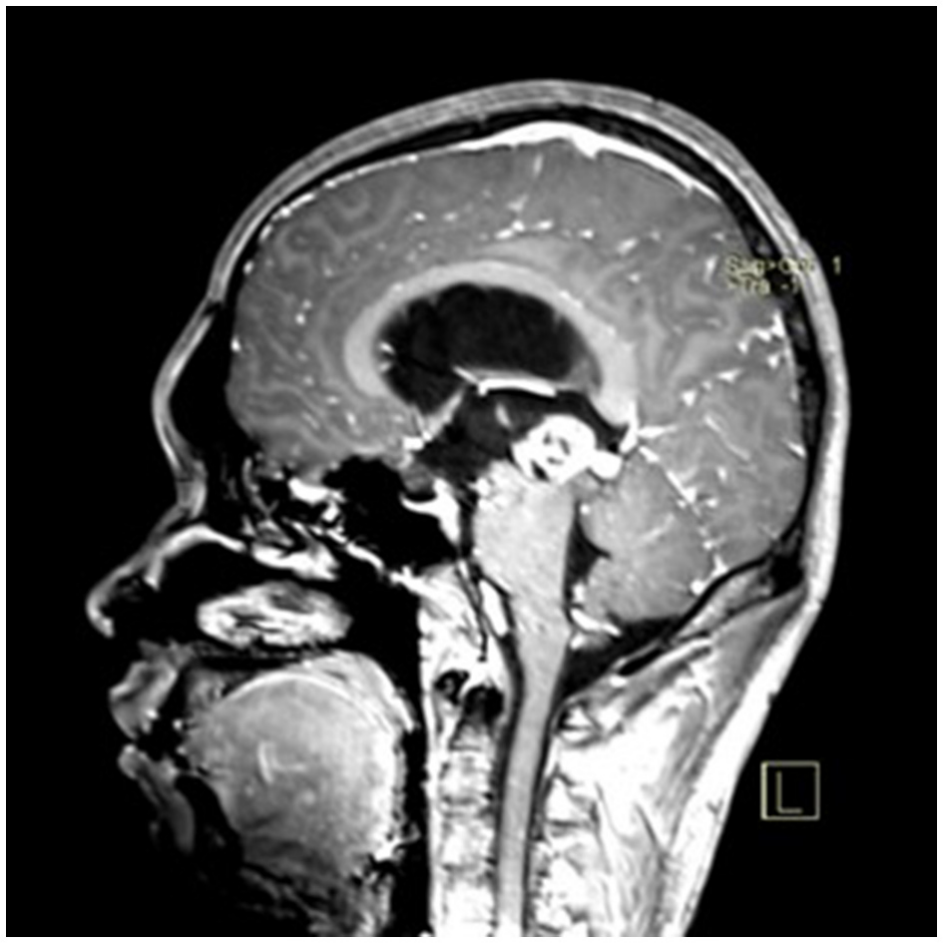
Preoperative contrast-enhanced MRI (sagittal view) showing the pineal region tumor.

**Figure 5:**
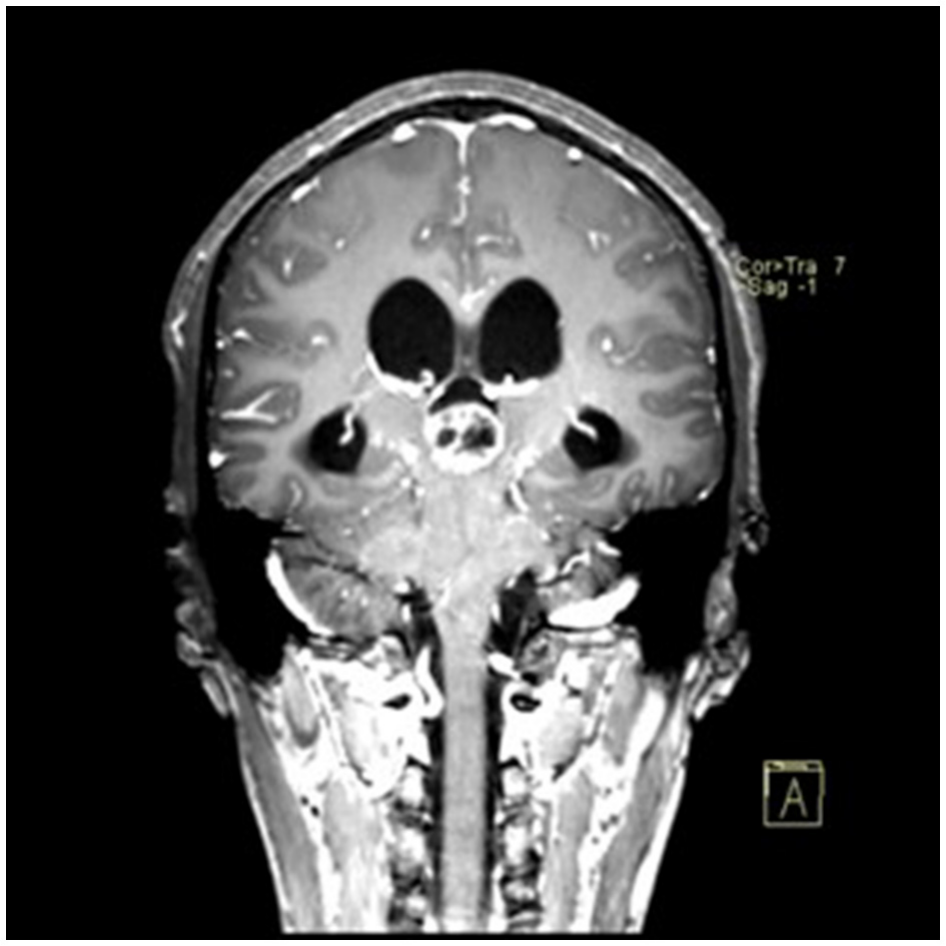
Preoperative contrast-enhanced MRI (coronal view) showing the pineal region tumor.

**Figure 6:**
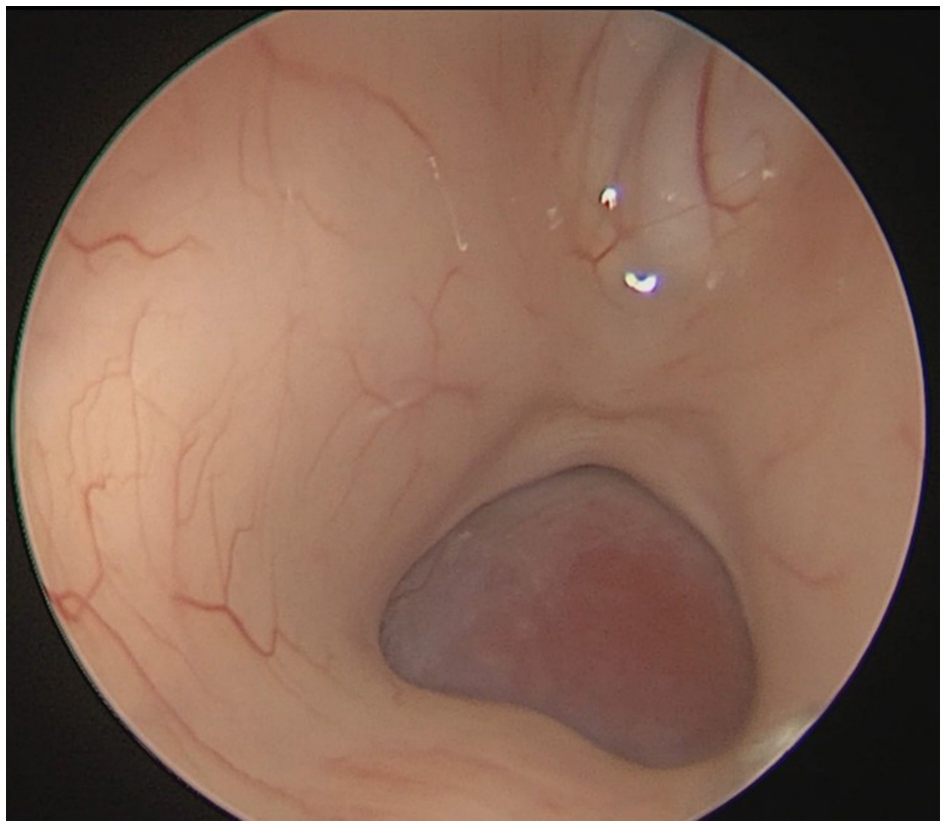
Intraoperative endoscopic view showing a ~20 × 20 mm tumor obstructing the cerebral aqueduct in the pineal region.

**Figure 7:**
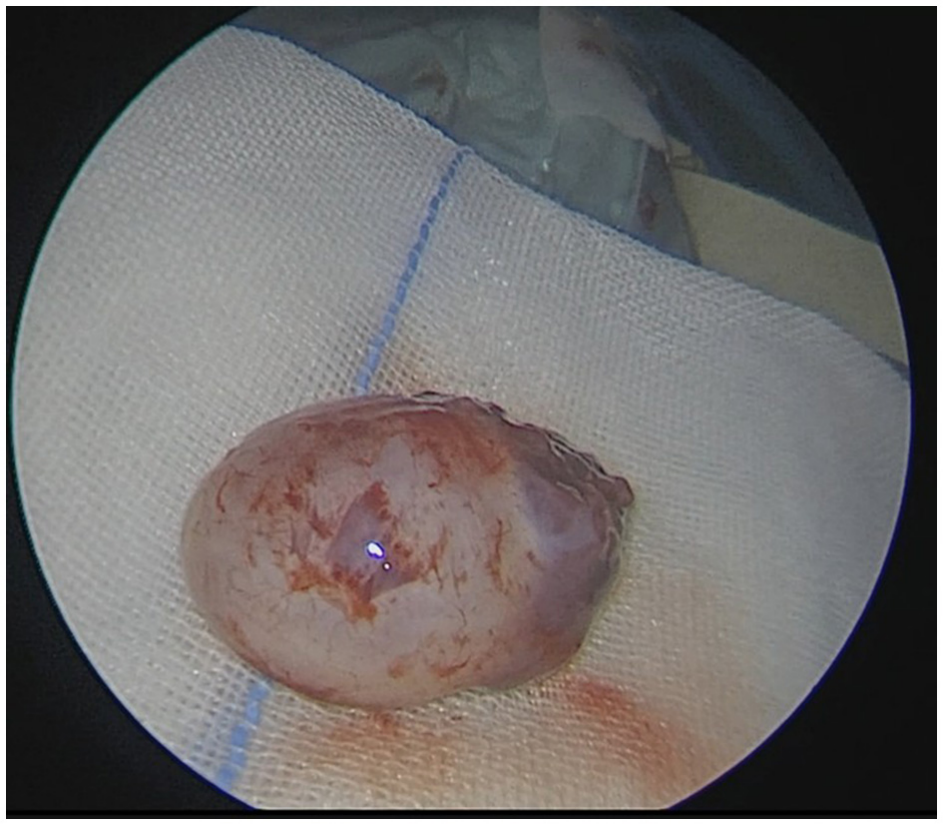
En bloc macroscopic resection of the tumor.

**Figure 8:**
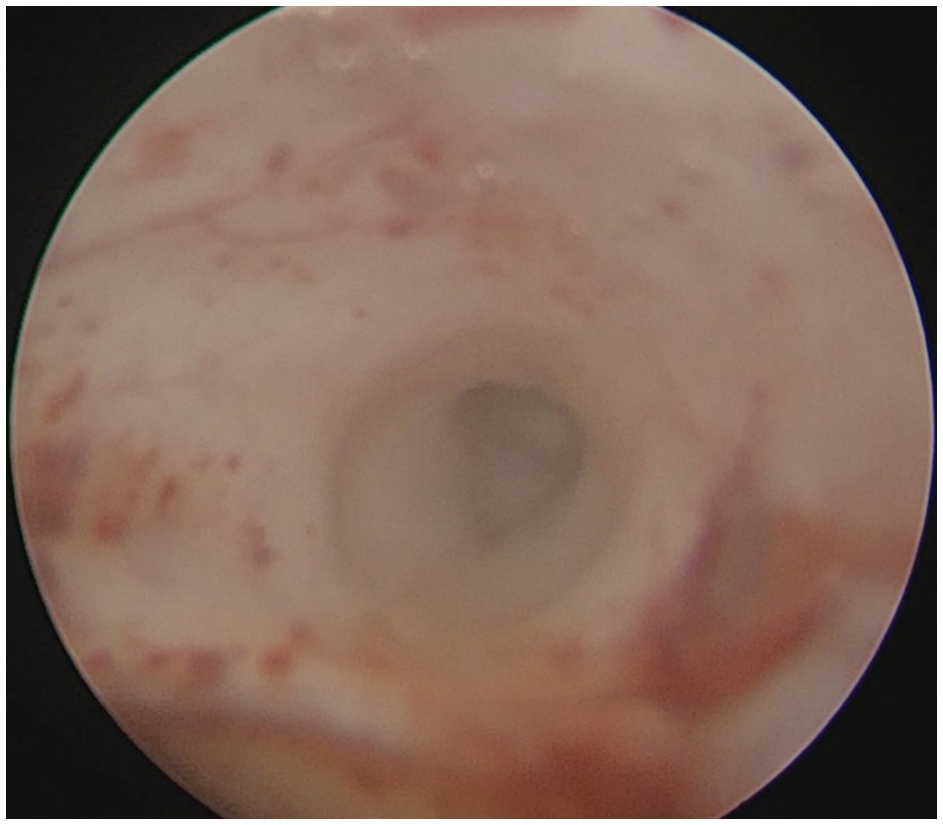
Endoscopic visualization confirming patency of the cerebral aqueduct.

**Figure 9:**
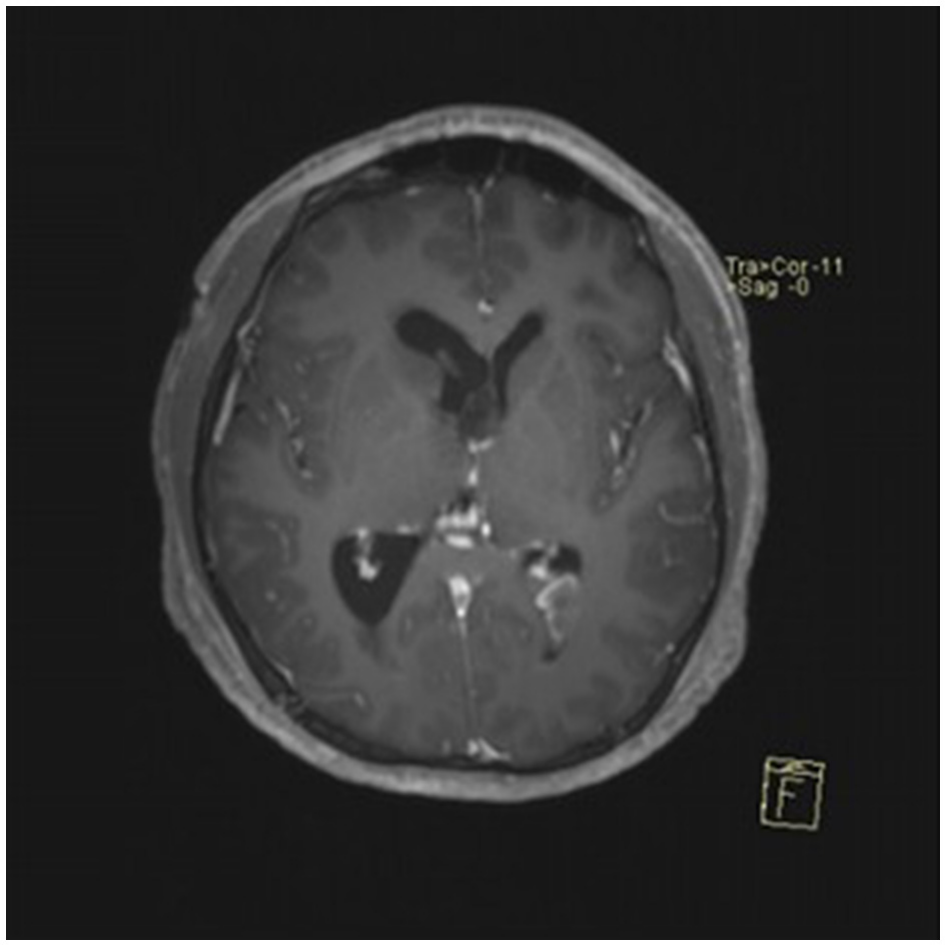
Postoperative day 1 contrast-enhanced MRI (axial view) showing complete tumor removal and resolution of hydrocephalus.

**Figure 10:**
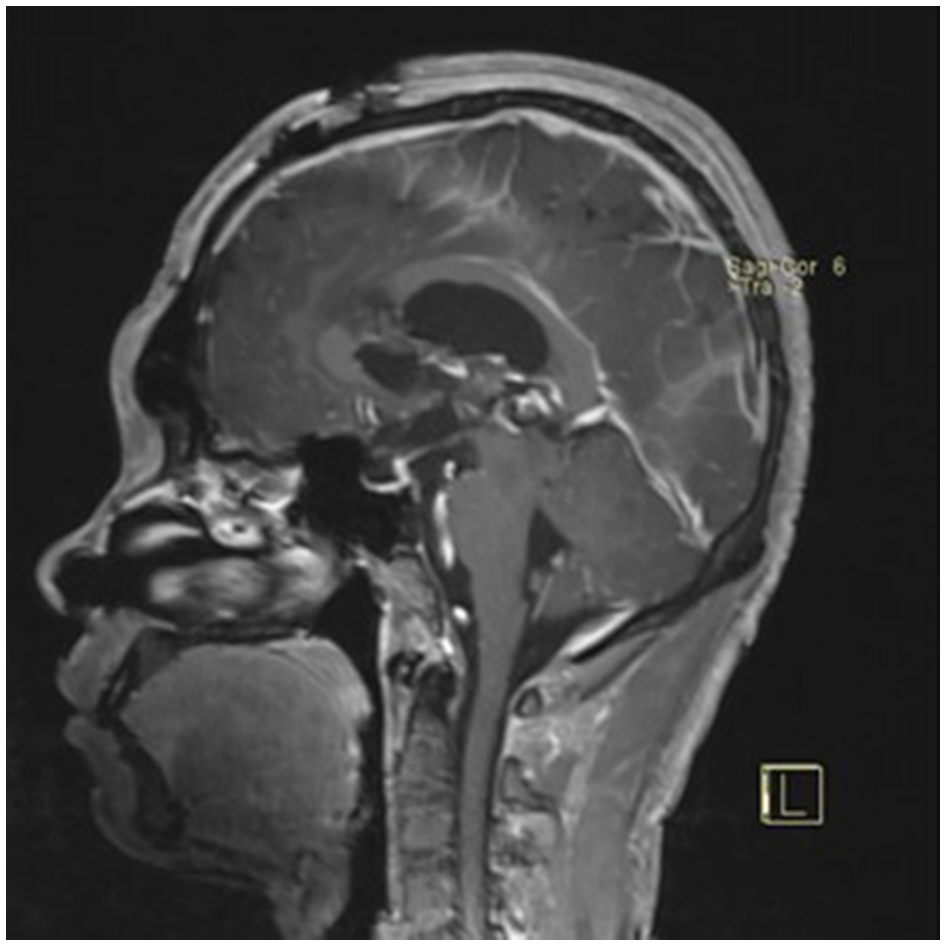
Postoperative contrast-enhanced MRI (sagittal view) showing no residual tumor.

**Figure 11:**
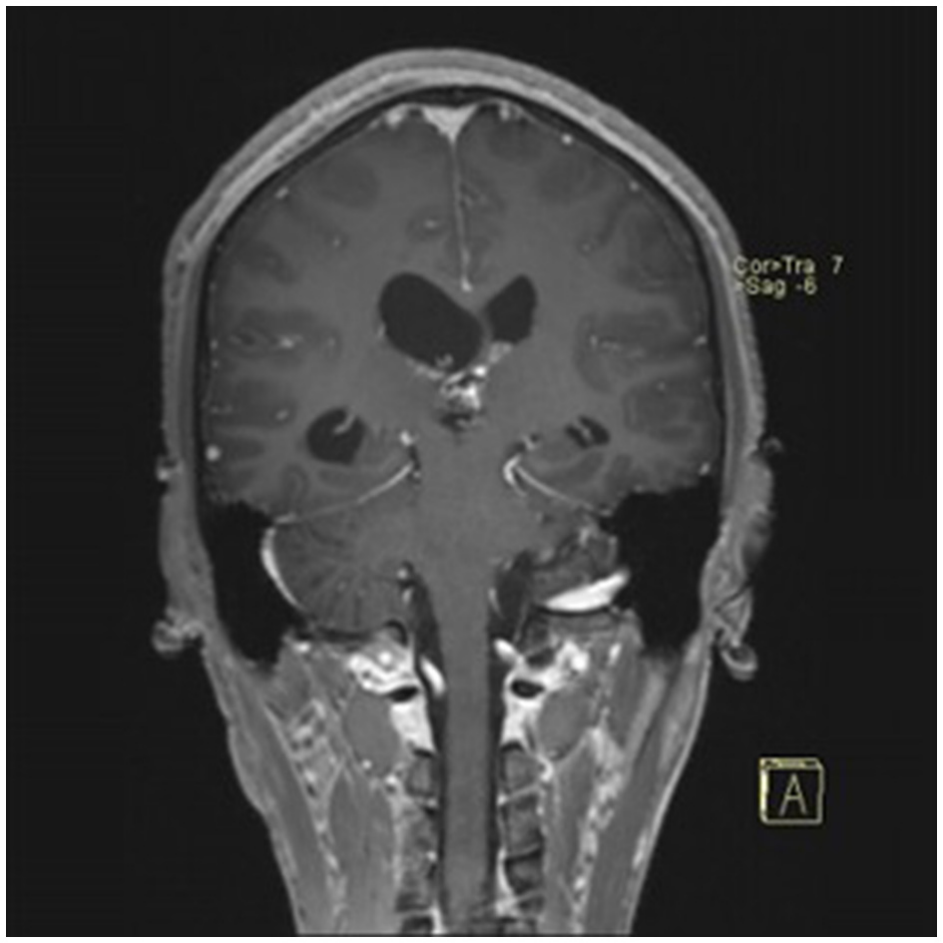
Postoperative contrast-enhanced MRI (coronal view) showing no residual tumor.

A 22-year-old male presented with intermittent dizziness and headache lasting for 2 weeks. MRI at admission revealed a cystic mass in the pineal region, accompanied by supratentorial hydrocephalus (Figures 3–5). No surgical contraindications were identified. After discussion with the patient's family, neuroendoscopic resection was performed via the Transfrontal-Transventricular-Transforaminal Approach. Intraoperatively, a 20 × 20 mm soft, well-vascularized tumor was found obstructing the cerebral aqueduct. The lesion had well-defined margins and was carefully dissected from surrounding adhesions, preserving adjacent veins and brain tissue. Macroscopic total resection was achieved en bloc (Figures 6–8). Postoperative day 1 MRI confirmed complete tumor removal and resolution of hydrocephalus (Figures 9–11). Postoperatively, no complications were observed. However, due to personal reasons, the patient was discharged on postoperative day 11. Histopathology confirmed a mixed germinoma. No recurrence or surgery-related complications were noted during follow-up.

## Discussion

Pineal region tumors are rare, accounting for 0.4–1% of adult intracranial tumors and 2.7–11% of pediatric intracranial tumors (1, 2). Radical resection is generally recommended for most pathological subtypes, except for radiosensitive entities, most notably germinomas, which are typically managed with biopsy followed by chemoradiotherapy and endoscopic third ventriculostomy (ETV). Studies show that radical resection offers better outcomes for nearly all benign pineal tumors and most malignant ones (3). However, the deep midline location of the pineal region and its diverse pathology make surgical resection highly challenging (4, 5).

### Neuroendoscopic surgery for pineal tumors

Given these surgical challenges, neuroendoscopic techniques have recently gained prominence in pineal tumor management due to their advantages in enhanced visualization, minimally invasive access, and high-resolution imaging (6, 7). Neuroendoscopic approaches include the supracerebellar infratentorial (Krause), occipital transtentorial (Poppen), intercallosal transfornicial, transfrontal-transventricular-transforaminal approach, ETV, and endoscopic tumor biopsy (ETB). The first three are adaptations of traditional microsurgical approaches enhanced by neuroendoscopy, with the Krause approach being the most common. However, each of these approaches has inherent limitations: the Krause route requires careful avoidance of deep venous structures such as the vein of Galen and provides limited exposure for tumors extending into the third ventricle; the Poppen approach offers a narrow operative corridor, making it difficult to access superiorly or laterally located lesions; and the intercallosal transfornicial approach necessitates traversing the corpus callosum and fornix, potentially increasing the risk of postoperative memory impairment (7–13).

### Anterior approaches to pineal region tumors via the foramen of Monro

The anterior neuroendoscopic approach through the foramen of Monro primarily includes ETV, ETB, and the transfrontal-transventricular-transforaminal approach. ETV effectively relieves obstructive hydrocephalus caused by pineal tumors, alleviating symptoms and reducing the need for permanent ventriculoperitoneal shunts (14–16). ETB reduces bleeding risks along the biopsy tract while providing adequate tissue for accurate pathological diagnosis. When pathology confirms pure germinoma, chemoradiotherapy can avoid the trauma and risks associated with craniotomy (14, 15). Several studies have reported successful single neuroendoscopic procedures combining tumor biopsy and ETV in pineal tumors, which reduce brain injury, complications, and hospital stay (14, 17–20). Nonetheless, postoperative bleeding remains a concern, as it may increase the risk of ETV failure and tumor dissemination (20).

The transfrontal-transventricular-transforaminal approach follows natural intracranial corridors, enabling safe access to the third ventricle without injuring adjacent neural or vascular structures. It is widely used for resection of third ventricle and periventricular lesions (21–25). Anatomical studies confirm excellent visualization of the posterior third ventricle and pineal region without interference from the vein of Galen complex, supporting its suitability for pineal lesions (23, 26–28). However, its application has primarily been limited to cyst fenestration, with few reports on solid tumors (21, 29–31). Our study represents the first case series to report the use of this approach for resection of pineal region tumors, demonstrating favorable outcomes.

### Key considerations for neuroendoscopic resection via the transfrontal-transventricular-transforaminal approach

Based on our experience, several key considerations should be emphasized: (1) Patient selection: Candidates with third ventricular hydrocephalus are preferred, as the foramen of Monro may enlarge up to 6-fold under such conditions, facilitating endoscopic maneuverability and reducing the risk of traction injury (28, 32). (2) Preoperative planning: Accurate preoperative neurosurgical navigation is essential for optimal placement of burr holes for tumor resection and ventriculostomy. The ideal entry point is typically located 5.2 cm above the supraorbital ridge and 2.5 cm lateral to the midline. (3) Intraoperative technique: Restoration of cerebrospinal fluid flow should be achieved by opening the mesencephalic aqueduct and performing a third or fourth ventriculostomy when necessary. For tumors adherent to surrounding tissues, sharp dissection is preferred. Partial tumor residuals may be acceptable to avoid damage to critical structures. (4) Hemostasis and protection: Ventricular wall injury should be minimized. Intraoperative bleeding should be aspirated promptly, and extravasation must be avoided to reduce the risk of postoperative hydrocephalus.

### Incision design and cosmetic considerations

The projection of the line connecting the anterior margin of the pineal region tumor and the foramen of Monro onto the anterior skull usually lies in the frontal region, where conventional incisions may leave noticeable scars. To achieve a better cosmetic outcome, we adopted an incision placed within the hairline. Although this approach requires a relatively larger incision and scalp flap, the scar can be concealed by hair regrowth postoperatively. Alternatively, two small frontal bone windows can be created separately for tumor resection and third ventriculostomy. This method allows for smaller incisions and faster healing but results in more visible scars that are difficult to conceal. The choice between the two approaches is made based on the patient's specific condition and personal preference.

### Advantages and highlights of this neuroendoscopic approach

The neuroendoscopic transfrontal-transventricular-transforaminal approach offers several benefits: (1) Compared with posterior approaches such as the supracerebellar infratentorial and occipital transtentorial routes, it avoids critical neurovascular structures by following natural ventricular corridors, sparing the vein of Galen, internal cerebral veins, and brainstem-adjacent structures, thus improving surgical safety. (2) For tumors extending from the pineal region into the third ventricle, this approach allows direct visualization of tumor-brain interfaces, enabling layer-by-layer “front-to-back, top-to-bottom” dissection aligned with tumor anatomy and growth. (3) If intraoperative assessment suggests inadequate restoration of ventricular drainage, third ventriculostomy can be performed immediately without repositioning, reducing postoperative hydrocephalus risk. (4) Surgeons are generally familiar with ventricular anatomy, making this approach straightforward, reproducible, and clinically promising. (5) Since all patients presented with hydrocephalus and foramen of Monro dilation, no significant operative limitations were encountered.

## Conclusions

This study introduces a neuroendoscopic approach that may represent a less invasive and safer alternative to previously reported techniques for pineal region tumor resection. However, several limitations must be acknowledged. Given the rarity of pineal region tumors, only eight patients met the inclusion criteria over a 5-year period at our institution, limiting the statistical power of the analysis. Additionally, as a single-center experience, the generalizability of our findings may be constrained. The relatively short follow-up period in some cases also limits long-term outcome assessment, given that this technique was recently adopted by our team. Larger, multicenter studies with longer follow-up are warranted to validate these findings.

In summary, neuroendoscopic resection of pineal tumors via the neuroendoscopic transfrontal-transventricular-transforaminal approach enables safe access to the posterior third ventricle while avoiding critical vascular structures, and represents an effective surgical option for selected cases.

## Data Availability

The original contributions presented in the study are included in the article/supplementary material, further inquiries can be directed to the corresponding author.
